# The effectiveness of automated digital health solutions at successfully managing obesity and obesity-associated disorders: A PICO-structured investigation

**DOI:** 10.1177/20552076221091351

**Published:** 2022-04-05

**Authors:** James Christopher Price, Heitor Oliveira Santos, Allain Amador Bueno

**Affiliations:** 1 College of Health, Life and Environmental Sciences, 8709 University of Worcester, UK; 2 School of Medicine, 28119 Federal University of Uberlândia (UFU), Uberlandia, Minas Gerais, Brazil

**Keywords:** digital health, smartphone applications, wearable technology, digital medicine offerings, obesity, hypertension, diabetes

## Abstract

Most adults in the UK and USA are classified as overweight or obese. Recent studies suggest that the prevalence of obesity has further increased during the SARS-CoV-2 pandemic and associated lockdowns. Digital technologies may be effective at managing obesity and related comorbidities, a potential further justified by social isolation and distancing circumstances.

This review of published literature employed a Patient-Intervention-Comparison-Outcome structured approach on the use of digital solutions to determine the effectiveness of their use in the management and treatment of obesity, hypertension, and type 2 diabetes and included commercially available, automated devices and applications that did not require intervention from a clinician. Our search covered studies published between January 2004 and February 2019, and 18 papers were included in the final analysis. The digital solutions reviewed were smartphone applications, wearable activity trackers, and ‘digital medicine offerings’ (DMO), including ingestible sensors and wearable patches.

This study found that not all interventions were effective at encouraging the lifestyle changes required for the management of obesity. Smartphone applications requiring interaction from the patient appeared to be more effective at encouraging engagement with treatment interventions than more passive wearable activity trackers. Automated feedback from smartphone applications was effective at managing type 2 diabetes, while DMO were effective at reducing blood pressure.

With the advancement of new technologies alongside a rapid increase in the prevalence of obesity and associated disorders, further studies comparing the various technologies available in larger sample populations for longer periods would help determine the most cost-effective preventive and therapeutic strategies.

## Introduction

In the UK, the majority of adults were classified as overweight or obese in 2017, with the country experiencing a huge increase in obesity since 1980.^1,2^ Recent studies show that following the SARS-CoV-2 pandemic, and the resulting lockdowns, obesity has become an even more pressing Public Health concern, not only in the UK but globally.^3–5^

Obesity is associated with significant ill health, with over 10,000 hospital admissions solely due to obesity and 711,000 admissions where it was a factor in the UK in 2017.^1^ Obesity is a major contributor to negative health outcomes in SARS-CoV-2 infection.^6,7^ As well as being a direct cause of ill health, obesity is often accompanied by other chronic conditions, such as hypertension and type 2 diabetes.^8^

Over 90% of adults in the UK and USA access the internet daily, with over 90% of adolescents and young adults having access to a smartphone.^9^ Due to their ubiquitous use, digital tools delivered via smartphones and through the internet are considered as a potential way of delivering interventions for the management of obesity and related disorders, among other medical conditions.^9^ Obesity, hypertension, and type 2 diabetes are similar in that they can be both ‘treated’ under the care of a clinical team or ‘managed’ by the patient themselves, such as by adjusting their lifestyle. Unfortunately however, it is widely accepted that poor adherence to healthier lifestyle interventions is a common occurrence in the longer term.^10^

Digital health solutions, such as mobile applications and wearable devices, have been recognised as a way to improve adherence to interventions that rely on self-monitoring and lifestyle changes, due to the fact that they stimulate constant interaction and, therefore, keep patients engaged with their treatment.^11^ Such self-monitoring has been particularly relevant in recent times due to the thoroughly justified need for self-isolation and the necessary quarantine measures during lockdown^12^

Hundreds of studies have been published in the last five years covering the usefulness of mobile applications and wearable devices in a range of health conditions. However, there is no consistency in the outcomes of such studies, and a more thorough understanding of the results available is necessary in order to further elucidate the potential of digital health solutions.

Although digital tools for self-monitoring are currently in use within the United Kingdom's National Health Service, their use is not currently widespread.^13^ The increasing popularity of commercially available digital health tools, such as smartphone applications and wearable activity trackers, offers an opportunity for existing patients, as well as overweight and obese individuals who are yet to present to a clinician, to monitor their condition daily, and make decisions based on this monitoring, without directly communicating with their physician. This is especially significant for patients who have not registered with a physician or who do not have health insurance, in remote settings where access to healthcare is difficult, or in situations where healthcare resources are diverted elsewhere, such as during the SARS-CoV-2 pandemic. Furthermore, it is estimated that the cost of obesity-related ill health to the National Health Service will rise from £6.1bn to £9.7bn a year by 2050,^14^ meaning that any effective digital solution that can improve obesity outcomes may allow this funding to be diverted to the treatment and management of other diseases.

Reviews into the treatment and management of obesity, hypertension, and type 2 diabetes have been carried out^15–17^; however, these studies do not focus on fully automated solutions. Given that such solutions involve interaction with a clinician, they are not scalable for use on a population level in the same way as fully automated solutions. The aim of this investigation was to explore the effectiveness of fully remote and fully automated digital health solutions in the treatment and management of obesity, hypertension, and type 2 diabetes.

## Method

**Figure 1.**
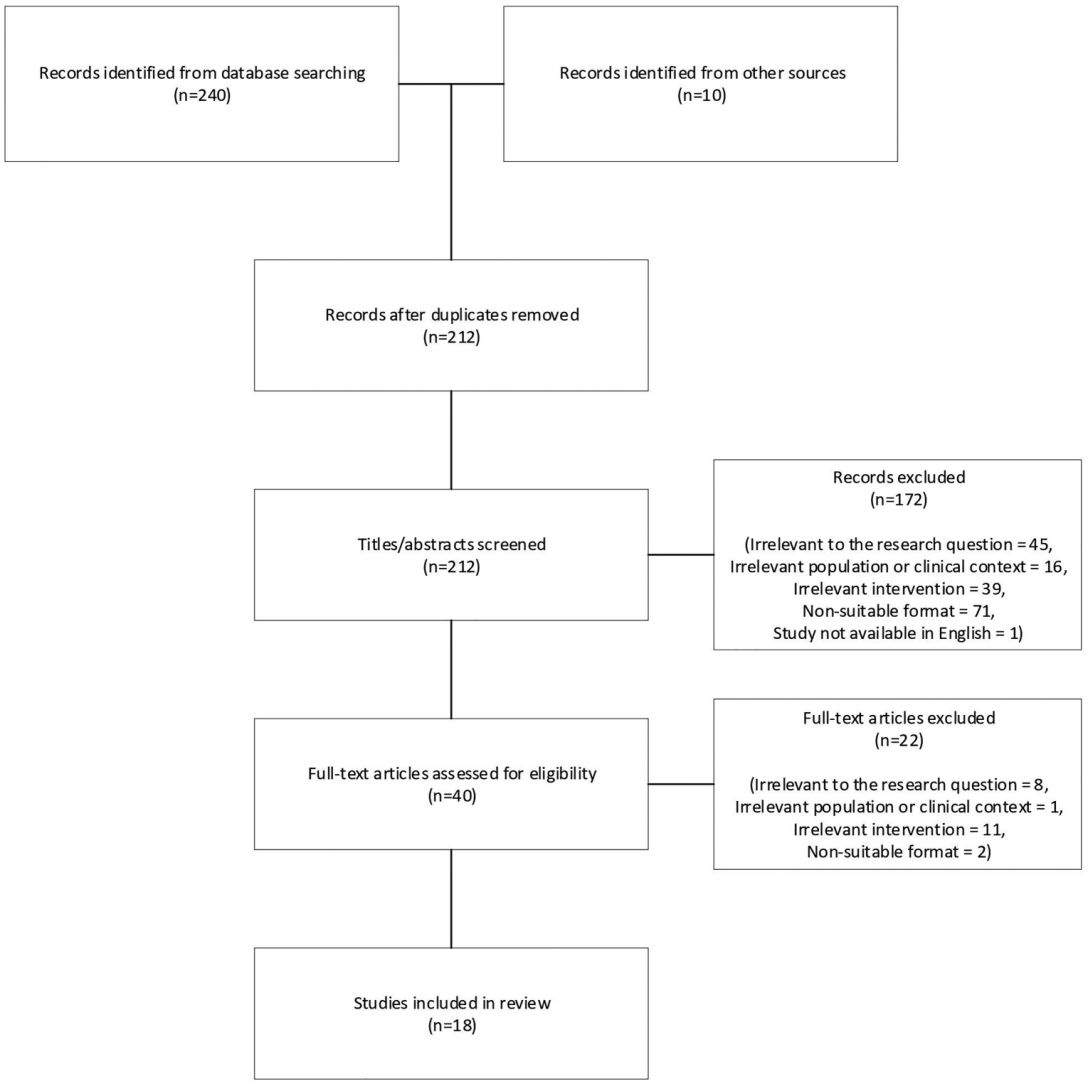
Flow chart diagram illustrating the search terms conducted and the final number of papers included in this literature review. Inclusion criteria detailed in the method. Search terms used for database searching can be seen in the appendix.

This literature review aimed to evaluate the current literature available on fully remote and fully automated digital health interventions and their use in managing obesity and related disorders. A Patient-Intervention-Comparison-Outcome (PICO) structured approach was used to frame the research question and is shown in Table 1. Figure 1 shows the Preferred Reporting Items for Systematic Reviews and Meta-Analyses (PRISMA) diagram for the searches carried out in this review. Searches were carried out in May 2021 on PubMed. PubMed was the only database searched, as this database contains journals that are indexed in the National Library of Medicine. Search results were screened by initially reviewing titles and abstracts by JP. Relevance criteria are explained below.

**Table 1.** The populations, interventions, comparisons, and outcomes studied in this literature review.

Population	Overweight and obese individuals Individuals with type 2 diabetes Individuals with hypertension
Intervention	Smartphone applications for daily self-monitoring Wearable activity trackers Digital medicine offerings (DMO)
Comparison	No treatment Non-digital interventions In-person interventions
Outcome	Weight loss Decrease in HbA1c Decrease in systolic or diastolic blood pressure

The initially selected papers were then fully reviewed by JP and HO and assessed for eligibility, with suitable and eligible papers included in the final review and shown in Table 2. No quality appraisal was carried out on the papers as this was outside the scope of this investigation. We instead focused on exploring the results of such studies to provide an overview of the literature available, rather than carrying out a meta-analysis.

**Table 2.** Literature review findings summarising digital health interventions aimed at treating or managing obesity, hypertension, or type 2 diabetes. Reference, population, intervention, comparison, outcome, and key findings are presented. Eighteen papers met the inclusion criteria.

Paper	Study format	Intervention details	Comparison details	Population characteristics	Sample size	Outcome measured	Relevant findings
Allen et al. 2013^18^	Randomised controlled pilot study	A smartphone application for daily self-monitoring which provided feedback, motivators, and the option of social support from others.	An established diet and exercise counselling intervention An established diet and exercise counselling intervention plus self-monitoring smartphone application A less intensive diet and exercise intervention with self-monitoring smartphone application	Overweight and obese adults (21–65-year-olds)	Started = 68, Completed = 43	Weight change at 6 months	The smartphone-only group experienced the least weight reduction, but no significant difference in weight reduction was observed between the four groups.
Bjørgaas et al. 2008^19^	Randomised controlled trial	A wearable activity tracker (pedometer) and encouragement to increase physical activity	Encouragement to increase physical activity	Patients with type 2 diabetes, some of whom were taking antidiabetic medication and insulin Intervention group baseline HbA1c = 7.4 mmol/mol (±1.1 mmol/mol) Comparison group baseline HbA1c = 7.7 mmol/mol (±1.4 mmol/mol)	Started = 70, Completed = 48	Change in weight, HbA1c and systolic and diastolic blood pressure after 6 months	No significant difference in changes in weight, HbA1c or systolic and diastolic blood pressure were observed between groups.
Carter et al. 2013^20^	Three-armed randomised controlled trial	A smartphone application that incorporates goal setting, daily self-monitoring, and automated feedback via text message	A commercially available website for daily self-monitoring Paper diaries for daily self-monitoring	Overweight volunteers	Started = 128, Completed = 79	Weight change at 6 months	Significantly greater weight reduction was seen in the smartphone group when compared with the website group, but not compared to the diary group.
Frias et al. 2017^21^	Three-arm cluster-randomised study	A DMO consisting of an ingestible sensor and wearable sensor patch, with a smartphone app to visualise the DMO data, alongside web portal for use by clinician, used for 4 or 12 weeks.	Usual care	Adults with uncontrolled hypertension and type 2 diabetes who had failed treatment (≥2 medications). Intervention group baseline HbA1c = 8.7% (±0.2%), baseline systolic blood pressure = 149.3 mmHg (±1.5 mmHg), baseline diastolic blood pressure 86.2 mmHg (±3.2 mmHg) Comparison group baseline HbA1c = 8.3% (±0.4%), systolic blood pressure 155.4 mmHg (±3 mmHg), baseline diastolic blood pressure = 83.9 mmHg (±2.9 mmHg)	Started = 118, Completed = 109	Change in systolic blood pressure (primary), change in glycated haemoglobin and proportion of participants reaching blood pressure goal (secondary)	In the 4-week group, DMO use resulted in a significantly greater reduction in SBP than usual care. This reduction was maintained at 12 weeks but was no longer significantly different from usual care. In the 12-week group, DMO use resulted in significantly greater reduction in SBP than usual care at 4 and 12 weeks. At 12 weeks, 98% of 12-week DMO participants achieved their blood pressure goal, compared to 51.7% of usual care participants. After 12 weeks, there was no significant difference in HbA1c reduction between groups.
Holmen et al. 2014^22^	Three-arm prospective randomised controlled trial	Blood glucose-measuring system with automated data transfer and a smartphone app with diet manual, and physical activity self-monitoring	The same intervention alongside health counselling from a diabetes specialist nurse Comparison group receiving normal care	Adults with type 2 diabetes (with an HbA1c of over 7.1%) Intervention group baseline HbA1c = 65 mmol/mol (±12.0 mmol/mol) Intervention + counselling group baseline HbA1c = 66 mmol/mol (± 12.0 mmol/mol) Comparison group baseline HbA1c = 67 mmol/mol (±13.1 mmol/mol) Medication status of participants was not reported	Started = 164, Completed = 120	Weight and HbA1c change after 4 and 12 months	There was no significant difference in change in HbA1c or weight between groups.
Kim et al. 2019^23^	Randomised controlled trial	A Bluetooth glucometer and smartphone application that calculated insulin dose from blood glucose readings	A group recording blood glucose levels using a paper logbook	Adults (19–80 years old) with HbA1c between 7.0% and 10.0% Intervention group baseline HbA1c = 7.7% (± 0.7%) Comparison group baseline HbA1c = 7.8% (± 0.7%) Participants continued to take any medication that had already been prescribed Mean baseline insulin was higher in the comparison group than the intervention group.	Started = 191, Completed = 151	Changes in HbA1c and proportion of participants whose HbA1c fell below 7% after 24 weeks	A significantly greater reduction in HbA1c and a significantly greater proportion of participants achieving a HbA1c level below 7% were observed in the mobile group than the paper diary group. Significantly more patients in the intervention group changed insulin dose compared to the comparison group.
Kim et al. 2019^24^	Three-arm randomised controlled trial	A smartphone app for daily self-monitoring and wearable activity tracker	Verbal advice to lose weight from a clinician	Patients with sleep apnoea	Started = 60, Completed = 43	Weight change after 4 weeks	Participants who used only the app experienced significant weight reduction. Participants that used the app and the wearable tracker experienced significant weight reduction. Comparison group participants did not experience significant weight reduction. App-only participants experienced significantly greater weight reduction than the comparison group, but app and wearable participants did not.
Kim, Wineinger & Steinhubl 2016^25^	Randomised controlled trial	A blood pressure monitoring device with smartphone application that gave reminders and health promotion material	Usual care	Hypertensive patients who had been prescribed at least one anti-hypertensive medication Intervention group baseline systolic blood pressure = 136.1 mmHg (±15.2 mmHg), baseline diastolic blood pressure = 86.3 mmHg (±12.8 mmHg) Comparison group baseline systolic blood pressure = 145.9 mmHg (±19.5 mmHg), baseline diastolic blood pressure = 93.1 mmHg (±14.1 mmHg)	Started = 95, Completed = 95	Changes in percentage of patients achieving blood pressure control systolic and diastolic blood pressure after 6 months	A significant decrease in diastolic blood pressure was observed in both groups, while a significant decrease in systolic blood pressure and increase in participants achieving blood pressure control were only seen in the comparison group.
Kooiman et al. 2018^26^	Randomised controlled trial	A wearable activity tracker and access to online health promotion resources	Usual care	Adults with type 2 diabetes (HbA1c ≥ 7.5%) taking insulin, oral medication, or GLP-1 therapy. No significant difference was observed for insulin usage between groups (intervention group = 55%, comparison group = 53%). Intervention group baseline HbA1c = 69.9 mmol/mol (±9.5 mmol/mol) Comparison group baseline HbA1c = 70.2 mmol/mol (±13.3 mmol/mol)	Started = 72, Completed = 66	Change in HbA1c after 13 weeks	No significant difference in change in HbA1c or change in medication was observed between groups
Mackillop et al. 2018^27^	Randomised controlled trial	A mobile phone-based solution and wireless blood glucose meter to monitor blood glucose levels during pregnancy	Routine clinic care	Women with gestational diabetes taking metformin Intervention group baseline HbA1c = 5.42% (±0.34%) Comparison group baseline HbA1c = 5.39% (±0.35%)	Started = 203, Completed = 183	Change in mean blood glucose level	No significant difference was observed for the change in mean blood glucose level between groups Changes in medication during the trial were not reported
Mameli et al. 2016^28^	Parallel arm randomised controlled trial	A smartphone application for daily self-monitoring of diet and a wearable activity tracker for automatic monitoring of energy expenditure	Instruction to follow a Mediterranean diet and limit sedentary behaviour	Obese 10–17-year-olds	Started = 43, Completed = 20	Weight change at 6 months	No significant difference in weight reduction was observed between the two groups.
Orsama et al. 2013^29^	Randomised controlled trial	A smartphone app for self-monitoring of weight, blood pressure and blood glucose, that provided automated feedback messages	Usual care	Individuals with a diagnosis of type 2 diabetes, elevated HbA1c levels, elevated systolic or diastolic blood pressure, or use of oral diabetes medication, between 30–70 years old. Intervention group baseline HbA1c = 6.86% (±1.56%), systolic blood pressure = 157.0 mmHg (±15.6 mmHg), diastolic blood pressure = 88.5 mmHg (±10.3 mmHg) Comparison group baseline HbA1c = 7.09% (±1.51%), systolic blood pressure = 146/5 mmHg (±15.3mmHg), diastolic blood pressure = 84.7 mmHg (±9.1 mmHg) Medication usage was not reported.	Started = 59, Completed = 48,	Changes in weight, HbA1c and blood pressure after 10 months	A significantly greater reduction in weight and HbA1c was observed in the intervention group compared to the comparison group. No significant difference between change in systolic or diastolic blood pressure was observed.
Patel et al. 2019^30^	Three-armed randomised controlled trial	A smartphone application to self-monitor diet for 12 weeks	An app to self-monitor weight and diet for 12 weeks and receive weekly lessons and feedback An app to self-monitor weight for four weeks and then diet and weight for 8-weeks	Overweight or obese adults	Started = 105, Completed = 100	Weight change at 3 months	There was no significant difference between weight reduction in the three arms and all arms resulted in clinically significant weight reduction.
Steinberg et al. 2013^31^	Randomised controlled trial	‘Smart’ scales, for daily self-weighing, that send results to a web portal, along with automated educational emails	A wait-list comparison group	Overweight and obese adults (18–60 years old)	Started = 91 Completed = 87	Weight change at 6 months	The intervention group lost significantly more weight than the comparison group and a significantly greater percentage of the intervention group achieved 5% weight loss
Svetkey et al. 2015^32^	Randomised, controlled comparative effectiveness trial	A smartphone application for daily self-monitoring that included goal setting, challenge games, automated reminders, and social support from other users	A smartphone assisted personal coaching intervention, where the self-monitoring was carried out on a smartphone app and goal setting, challenges, and social support were delivered in person by a dietitian, along with monthly calls A comparison group who received informational leaflets but were not asked to self-monitor	Overweight or obese 18–35-year-olds	Started = 365, Completed = 313	Weight change at 6, 12 and 24 months	No significant difference in weight reduction was seen between any of the groups over 24 months. The personal coaching group experienced greater weight reduction than the app-only group and significantly greater weight reduction than the comparison group at 6 months.
Tudor-Locke et al. 2004^33^	Randomised controlled trial	Wearable activity tracker (pedometer)	A wait-list comparison group	Obese adults	Started = 60 Completed = 47	Change in weight, systolic and diastolic blood pressure, and glycated haemoglobin at 16 weeks.	No significant difference was seen between the changes in any outcome in each group. No significant reduction in any outcome was observed in either group.
Waki et al. 2014^34^	Randomised controlled trial	Smartphone app for daily self-monitoring of diet, weight, blood glucose and blood pressure, as well as automated feedback messages about diet input	Continuation of ‘self-care regimen’	Individuals with type 2 diabetes Average baseline HbA1c values were not reported Intervention group medication usage: 7/27 were taking no medication, 13/27 were taking oral hypoglycaemic alone, 4/27 were taking injectable noninsulin alone, 3/27 were taking injectable noninsulin and oral hypoglycaemic Comparison group medication usage: 6/27 were taking no medication, 20/27 were taking oral hypoglycaemic alone, 0/27 were taking injectable noninsulin alone, 1/27 were taking injectable noninsulin and oral hypoglycaemic	Started = 66, Completed = 54	Change in HbA1c after 3 months	A significantly greater reduction in HbA1c was seen in the intervention group compared to the comparison group. No significant difference in medication adjustment was observed between groups.
Yoo et al. 2009^35^	Randomised controlled trial	A blood glucose measuring device, blood pressure measuring device, mobile phone with automated alerts to take and upload measurements	Conventional clinic visits	Overweight individuals, aged 30–70, with diagnosed hypertension and type 2 diabetes Intervention group baseline HbA1c = 7.6% (±0.9%), systolic blood pressure = 140 mmHg (±18 mmHg), diastolic blood pressure = 84 mmHg (±10 mmHg) Comparison group HbA1c = 7.4% (±0.9%), systolic blood pressure = 138 mmHg (±18 mmHg), diastolic blood pressure = 83 mmHg (±10 mmHg) Medication usage was not reported.	Started = 123, Completed = 111	Change in HbA1c, blood pressure and weight after 3 months	A significant reduction in systolic and diastolic blood pressure was observed in the intervention group but not in the comparison group. A significant reduction in HbA1c was observed in the intervention group compared to a significant increase in HbA1c in the comparison group. A significant decrease in weight was observed in both groups.

Search terms resulted in a total of 240 studies and 10 studies were identified from other sources, with 18 studies included in the final review. Studies were not excluded based on the year of publication and the included studies were published between 2004 and 2019. Separate searches were carried out for obesity, hypertension, and type 2 diabetes, with relevant interventions and outcomes included in each search. For example, “glycated haemoglobin” was only included in type 2 diabetes search term, and “digital medicine offering” (DMO) was not included in the obesity search term as it would not be typical for obesity to be treated with such an intervention in the same way as hypertension or type 2 diabetes. The search terms employed in this study are presented in the appendix.

‘Digital health’ is a broad term which can cover a wide range of interventions. A study was included in this review if it involved: a) the use of mobile or web-based applications for self-monitoring of diet or exercise; or b) the use of wearable activity trackers; or c) the use of digital tools which are used by the patient to self-monitor physiological factors, such as digital blood pressure (BP) monitors and blood glucose monitors; or d) DMO, which are defined in this study as a prescribed medication that is enhanced by technology, such as ingestible sensors and wearable insulin delivery devices.

Only digital interventions which were fully automated and did not involve remote or in-person contact with healthcare professionals were included in this study. This approach was so to ensure that all the interventions reviewed here were fully scalable and could be used on a population level. Studies that included commercially available and bespoke applications were included if they did not involve contact with healthcare professionals, on the basis that these bespoke applications could be distributed on a large scale or commercialised and released to the public. Although studies were excluded if they included contact with a healthcare professional during the study, many of the appraised studies took place in healthcare professional settings for data collection purposes and, therefore, included meetings with healthcare professionals at the start and end of their interventions.

Studies that included digital tools that alerted healthcare practitioners if the patient recorded dangerous blood glucose or BP reading were included in this review, as the main function of these applications was to self-monitor the condition, with the alerts acting as an additional safety feature, with these alerts not affecting the way that the patient can self-monitor their condition. Furthermore, interventions that sent BP and blood glucose readings to healthcare practitioners but did not involve regular remote contact with these healthcare practitioners were included. This is because if a patient is managing hypertension or type 2 diabetes following a diagnosis, they are, by definition, already under the care of a healthcare practitioner. Therefore, the provision of their data to the healthcare practitioner may benefit their overall treatment plan but does not impact the way that they use the digital health tool to manage their condition on a day-to-day basis. Interventions in the included studies were compared against comparison groups defined by the authors of the studies and are listed in the ‘Comparison Details’ column of Table 2.

Only studies that included primary data (e.g. randomised control trials) were included in this investigation. Review papers were excluded. Where review papers appeared to be relevant to the topic, their reference lists were analysed, and relevant papers found were included in the review. These papers represent the ‘records identified from other sources’ on the PRISMA diagram. Only articles written in English were included.

## Results and discussion

### Obesity

Weight reduction was an outcome investigated in 11 of the studies included in this literature review,^2,18–20,22,28–33^ with some studies investigating weight reduction alone^18,20,22,24,28,30–32^ and some investigating it alongside outcomes relevant to comorbidities such as hypertension and diabetes.^19,29,33^ Smartphone applications were included in nine of these studies^18,20,22,24,28–31,33^ and were the most common digital intervention studied, followed by wearable activity trackers,^19,24,28,33^ which were included in four of the studied interventions.

Smartphone applications that involved daily self-monitoring of diet, exercise, or weight were found to be effective at reducing weight in users in all of the studies appraised in this review, apart from Mameli et al.,^28^ which included children as young as 10 years old who may not have total agency over their diets. Allen et al*.*^18^ found that weight reduction in individuals using a daily self-monitoring smartphone application, with automated motivational feedback messages, was not significantly different from weight reduction in an in-person diet and exercise counselling intervention. However, the smartphone-only group did lose the least weight overall and the authors note that, since it is a pilot study, it is not sufficiently powered to detect significance between the groups.

Carter et al.^20^ found similar outcomes for weight reduction between two groups using a smartphone application or paper diaries for self-monitoring, suggesting that it is the action of engaging with the self-monitoring on a daily basis that drives the behaviour change required for successful weight reduction. The smartphone and paper diary groups were also compared to a group using a website for daily self-monitoring, and significantly less weight reduction was seen in this group than in the smartphone group. The authors suggest that those results could be explained by the fact that the participants were used to using their smartphones on a daily basis, meaning the introduction of daily self-monitoring into their lives was less of an intrusion.

Furthermore, daily self-monitoring included diet, physical activity, and weight in most of the studies in this review, suggesting that adherence to a calorie-restricted diet was made more successful by the ability to track the diet on a smartphone. Interestingly, Steinberg et al.^31^ found that daily self-monitoring of only weight resulted in significantly more weight reduction than a waitlist comparison group. This suggests that the process of monitoring weight daily, and thus keeping the goal of weight reduction in the mind, was enough to drive the behaviour change required to reduce weight. This is supported by the fact that participants in Steinberg's study consumed fewer calories than the comparison group, despite not being told to self-monitor diet. However, given that study compared the intervention group to a waitlist comparison group, rather than usual care, these results only demonstrate that measuring weight daily results in better outcomes than simply not measuring it.

The use of wearable activity trackers was generally not found to be associated with more weight reduction by the studies included in this review. Bjørgaas et al.^19^ found that the use of a pedometer alongside advice to increase physical activity did not result in significantly more weight reduction than the advice on its own, suggesting that wearable trackers alone are not an effective way of achieving weight reduction. Tudor-Locke et al.^33^ reported similar findings, that the use of a pedometer did not result in significantly more weight reduction than a waitlist comparison group. Interestingly, participants in Tudor-Locke's study using the pedometer did significantly increase their physical activity compared to the comparison group, suggesting that the participants were overestimating the calories expended during this physical activity.

Ultimately, long-term adherence to low-calorie diets, irrespective of dietary patterns, remains a core contributor to weight loss, overpowering the beneficial effects from physical exercise alone.^36^ Nevertheless, several RCTs and observational studies demonstrate the importance of both exercise and physical activity simultaneously in reducing the risk for obesity and major adverse cardiac events due to, for example, increased daily steps, which is a parameter that can be reliably quantified by the digital tools explored in this review.^37–39^

Kim et al.^24^ found that a combination of a smartphone application for daily self-monitoring and wearable tracker was associated with significant weight reduction. When compared with results reported by Bjørgaas and Tudor-Locke, Kim's results suggest that the use of the wearable tracker in combination with a smartphone allows the user to track the calories that have been expended by physical activity more accurately and adjust their diets accordingly, something that could not have been identified in Bjørgaas’ and Tudor-Locke's studies, as they are from before smartphone use was widespread. Kim et al.^24^ also report that participants that used the smartphone application without the wearable tracker experienced significantly more weight reduction than the comparison group, which received verbal advice to lose weight, whereas the group that used both the application and the wearable did not.

Many of the studies that were excluded from this review included smartphone-based interventions that combined daily self-monitoring with remote contact with a clinician or in-person interventions assisted by smartphones. These studies were excluded from our investigation as they are not fully automated, but this type of intervention was included as a comparison in two of the studies included in this review.^30,32^ Svetkey et al*.*^32^ reported no significant difference in weight reduction between groups using a smartphone application with a gamification aspect and receiving an in-person coaching intervention assisted by a smartphone, after 24 months, while the in-person group had greater weight reduction than the smartphone group after 6 months. Such observations suggest that, over a longer period, smartphone-based interventions may be as effective as in-person interventions supported by smartphones. Furthermore, Patel et al.^30^ found no significant difference in weight reduction between a group using a smartphone application for daily-self monitoring and another group using the same application alongside weekly lessons and feedback, with both groups experiencing clinically meaningful weight reduction.

### Hypertension

Hypertension-related outcomes, such as reduction in systolic and diastolic BP and the percentage of participants achieving their BP target, were investigated by six of the studies included in this review.^19,21,25,29,33,35^ Smartphone applications were included in four of the studies,^21,25,29,35^ with wearable trackers being included in three^19,21,33^ and DMO included in one.^21^

Frias et al*.*^21^ investigated the effectiveness of a DMO intervention involving a smartphone application, ingestible sensor, and wearable sensor patch in adults who had failed treatment for hypertension and type 2 diabetes, compared to usual care. They found that systolic BP reduction was significantly greater in the DMO group than the usual care group after 4 weeks and 12 weeks. Furthermore, participants that only used the intervention for 4 weeks maintained their reduction in systolic BP at 12 weeks, with outcomes at this time being comparable with usual care, suggesting that shorter term use of DMO interventions may have lasting benefits.

Yoo et al*.*^35^ studied an intervention that included participants manually recording their BP and uploading it, with automated feedback messages reminding them to measure and upload and also providing insight as to whether their readings were high. Participants using this intervention achieved a significant reduction in systolic and diastolic BP, compared to the comparison group, who attended conventional clinic visits and interestingly did not achieve any significant change. The intervention studied by Orsama et al*.*^29^ also involved a smartphone application for daily self-monitoring that included feedback messages and was found to be as effective as usual care at reducing systolic and diastolic BP.

The intervention studied by Kim et al.^25^ followed a similar approach, but the BP readings were automatically uploaded via wireless connectivity with a smartphone application. This intervention was compared to usual care as a comparison group. The authors found that both groups achieved a significant decrease in diastolic BP, but only the usual care group achieved a significant decrease in systolic BP. Furthermore, only the usual care group had an increase in the percentage of participants achieving BP control. These findings support the suggestion that the act of manually recording and uploading self-monitoring readings may be instrumental in driving the behaviour change required to achieve meaningful outcomes while using digital interventions and that the act of daily self-monitoring may result in the patient better adhering with their required lifestyle changes. This is further supported by the fact that the intervention was more effective in participants with ‘patient activation’, a concept that measures how confident and engaged patients are with managing their condition. Furthermore, it is worth speculating that patients in the usual care group were asked about their medication by their healthcare professional, which may have improved medication adherence and contributed to the increased percentage of participants achieving BP control. Further research into whether interventions with automated medication reminders are effective would provide evidence as to whether such reminders are effective at improving outcomes.

Bjørgaas et al.^19^ and Tudor-Locke et al.^33^ investigated the use of wearable trackers on their own at reducing systolic and diastolic BP. As was the case for weight reduction, neither study found evidence that wearable trackers alone are an effective way of managing hypertension. Bjørgaas et al.^19^ found no significant difference between the intervention group and a group that was encouraged to increase their physical activity, while Tudor Locke et al.^33^ found no significant difference between the intervention group and a waitlist comparison group.

### Type 2 diabetes

Glycated haemoglobin (HbA1c), one of the primary metrics for diagnosing and measuring the extent of type 2 diabetes, is included as an outcome in ten of the studies included in our investigation. Smartphone applications for self-monitoring were included in seven, wearable activity trackers in four, and DMO in four studies.

As was the case for obesity and hypertension, Bjørgaas et al.^19^ and Tudor-Locke et al.^33^ did not find evidence that the use of a wearable activity tracker was more effective than advice to increase physical activity^19^ or than a waitlist comparison group^33^ at reducing HbA1c. Kim et al.^23^ report on an intervention in which participants used a smartphone application for daily self-monitoring, as well as a Bluetooth-enabled glucometer that feeds data to the application, allowing the application to calculate insulin dose from blood glucose readings. Kim and colleagues found that participants using this intervention achieved a significantly greater reduction in HbA1c compared to a comparison group using a paper logbook to self-monitor blood glucose, as well as a significantly greater proportion of participants reducing HbA1c to under 7%. In another study involving an intervention that automatically uploaded blood glucose readings, Holmen et al.^22^ found that participants using this intervention experienced a change in blood glucose that was not significantly different from the change seen in a comparison group receiving usual care or a group using the intervention alongside counselling from a diabetes nurse.

Kooiman et al.^26^ found that the use of a wearable activity tracker and access to online health promotion resources resulted in a change in HbA1c that was not significantly different from the change found in a usual care comparison group. Furthermore, Mackillop et al.^27^ found that participants using a smartphone app for daily self-monitoring of blood glucose resulted in a change in HbA1c that was not significantly different from a comparison group receiving routine clinical care. In a study that compared a digital intervention to a self-care regimen, Waki et al.^34^ reported that participants using a smartphone application for daily-self monitoring with automated feedback messages achieved a significantly greater reduction in HbA1c than the comparison group. Orsama et al.^29^ also measured the effect of their intervention, which included feedback messages, on HbA1c reduction and found that it resulted in a significantly greater reduction than usual care, with similar results reported by Yoo et al.^35^

Frias et al.^21^ found that the use of an ingestible sensor, wearable sensor patch and smartphone application for daily self-monitoring resulted in a reduction in HbA1c that was not significantly different from usual care. That DMO intervention was found to result in a greater reduction in systolic and diastolic BP than usual care, but a similar reduction in HbA1c. This could be because hypertension treatment is likely to involve some form of oral medication and the DMO is thought to be effective primarily by monitoring and improving medication adherence.

### Overall analysis and final considerations

This Patient-Intervention-Comparison-Outcome-structured investigation found that the effectiveness of digital health tools at managing and treating obesity, hypertension, and type 2 diabetes is variable and that some digital interventions were more effective than others. The use of wearable activity trackers, without smartphone application integration, were found to be as effective as a waitlist comparison group,^33^ or as verbal encouragement, to increase physical activity^19^ aiming at improving obesity-, hypertension-, or diabetes-related outcomes. We also found that when such interventions were combined with access to online educational material, they induced reductions in HbA1c similar to those found through regular visits to a specialist diabetes nurse,^26^ suggesting that wearable technology can be effective if used as part of a broader digital intervention with access to educational material.

Combining a wearable activity tracker with a smartphone application was found to result in a significantly greater reduction than verbal advice to lose weight by one study in this review.^24^ The same study found that the use of the smartphone application on its own was more effective than in combination with the wearable tracker, suggesting that the use of the wearable tracker may have caused users to overestimate the calories expended by their increased physical activity.

Smartphone applications were the most commonly studied intervention in our study, and applications that involve daily self-monitoring of diet and physical activity were found to be as effective as usual care at reducing weight by the majority of the studies appraised. Furthermore, using a portable device that the user is familiar with may be more effective than a web-based portal accessed via a computer. These interventions were also found to be effective at reducing HbA1c, especially when integrated with a glucometer allowing a calculated insulin dose to be fed back in real time. Applications that provided feedback in real time were found to be more effective at managing diabetes than hypertension, compared to usual care, suggesting that the feedback messages are more useful to diabetes patients who control their own medication doses, than hypertension patients, who are likely to be prescribed specific doses of medication. Many of the self-monitoring applications appraised in our study involved the patient simply measuring and recording their HbA1c or BP values and yet reduction in these values was observed. This suggests that the act of monitoring these values results in the patient changing their behaviour and motivates them to adhere to the lifestyle changes required to effectively manage their condition. Our suggestion is further supported by the effectiveness of automated feedback messages, which not only remind the patient to record their HbA1c and BP values but also improve outcomes by allowing more intelligent calculation of insulin dosage and daily calorie consumption.

DMO, such as a solution that integrated an ingestible sensor, wearable patch, and smartphone application, were found to result in significantly greater reductions in systolic and diastolic BP than usual care, while inducing similar reductions in HbA1c as compared to usual care.^21^ Given that the ingestible sensor and wearable patch are primarily for monitoring and improving adherence to medication, these findings suggest that DMO are more effective at managing hypertension than type 2 diabetes, possibly because hypertension patients are more likely to have oral medication, whereas type 2 diabetes patients are more likely to be delivering insulin.

Treating or managing health conditions with wearable devices alone and omitting conventional treatments which are known to be effective, such as pharmacological treatments, is ethically questionable. However, given that obesity is a condition that can be managed with behavioural change alone, it is a good candidate for research into whether devices alone could improve disease outcomes without external interference.

## Conclusion

Overall, the findings of this investigation suggest that smartphone applications for self-monitoring of diet, physical activity, and weight are effective at inducing the behavioural change required to reduce weight, BP, and HbA1c. Non-digital self-monitoring interventions can produce similar outcomes, but the effectiveness of digital interventions is enhanced when they include automated feedback to patients, especially for type 2 diabetes patients where feedback messages include insulin doses. DMO are primarily aimed at monitoring medication adherence and, therefore, appear to be more effective when used by hypertension patients who have oral medication as part of their treatment. Wearable activity trackers do not appear to be effective at reducing weight, BP, or HbA1c on their own, but may be effective as part of a wider intervention, especially if these trackers provide accurate estimates of calorie expenditure.

Given that fully automated interventions benefit from the fact that they can be scaled to the population level, long-term studies in large free-living sample populations would be extremely helpful in determining the effectiveness of the potentially preventive and therapeutic benefits of digital health applications, their cost-effectiveness and feasibility compared to conventional treatments. If such interventions were to be scaled to the population level, they would likely be used by individuals who are undergoing treatment for disease, where the automated digital solution would be delivered in combination with conventional treatment, as well as for those in the very early stages of diabetes, obesity, and hypertension, who are yet to be prescribed medical intervention. Further research into how conventional, in-person treatment and automated digital tools can be combined to treat patients would provide insight into how this would affect the efficacy of these treatments. Furthermore, research into how these interventions can prevent the progression of obesity, hypertension, and type 2 diabetes or result in early diagnosis would provide insight into the potential economic benefits that these solutions could bring to health services.

The preventive and therapeutic potential of such interventions is further justified by the pressing need to tackle the continuously increased prevalence and incidence of obesity in the past decades, markedly accentuated in the past two years due to the pandemic and lockdowns. Added to that is the need for interventions that follow social distancing measures necessary to reduce the transmission of SARS-CoV-2. Lastly, investigations into the attitudes towards digital health of patients from different demographics would provide a better understanding of any barriers to their use or inequalities that exist when they are used.

## Appendix

Search terms used for initial database searching:

(“smartphone application” OR “mobile application” OR “wearable tracker” OR “wearable activity tracker” OR “digital health” OR “digital intervention”) AND (“weight loss” OR “weight reduction” OR BMI OR obesity OR overweight) AND (“RCT” OR “controlled trial” OR randomised OR random)

(“smartphone application” OR “mobile application” OR “wearable tracker” OR “digital medicine” OR “digital medic*” OR “DMO” “wearable activity tracker” OR “digital health” OR “digital intervention”) AND (“hypertension” OR “blood pressure”) AND (“RCT” OR “controlled trial” OR randomised OR random)

(“smartphone application” OR “mobile application” OR “wearable tracker” OR “wearable” OR “digital medicine” OR “digital medic*” OR “DMO” “wearable activity tracker” OR “digital health” OR “digital intervention”) AND (“diabetes” OR “diabetic” OR “glycated haemoglobin” OR “hemoglobin” OR “haemoglobin” OR “HbA1c”) AND (“RCT” OR “controlled trial” OR randomised OR random)
